# The Associations of Uric Acid, Cardiovascular and All-Cause Mortality in Peritoneal Dialysis Patients

**DOI:** 10.1371/journal.pone.0082342

**Published:** 2014-01-08

**Authors:** Jie Dong, Qing-Feng Han, Tong-Ying Zhu, Ye-Ping Ren, Jiang-Hua Chen, Hui-Ping Zhao, Meng-Hua Chen, Rong Xu, Yue Wang, Chuan-Ming Hao, Rui Zhang, Xiao-Hui Zhang, Mei Wang, Na Tian, Hai-Yan Wang

**Affiliations:** 1 Renal Division, Department of Medicine, Peking University First Hospital; Institute of Nephrology, Peking University; Key Laboratory of Renal Disease, Ministry of Health; Key Laboratory of Renal Disease, Ministry of Education; Beijing, China; 2 Department of Nephrology, Peking University Third Hospital, Beijing, China; 3 Department of Nephrology, Huashan Hospital of Fudan University, Shanghai, China; 4 Department of Nephrology, Second Affiliated Hospital of Harbin Medical University, Heilongjiang, China; 5 Kidney Disease Center, The First Affiliated Hospital, College of Medicine, Zhejiang University, Hangzhou, China; 6 Department of Nephrology, Peking University People's Hospital, Beijing, China; 7 Department of Nephrology, General Hospital of Ningxia Medical University, Ningxia, China; Shanghai Institute of Hypertension, China

## Abstract

**Aims:**

To investigate whether uric acid (UA) is an independent predictor of cardiovascular (CV) and all-cause mortality in peritoneal dialysis (PD) patients after controlling for recognized CV risk factors.

**Methods:**

A total of 2264 patients on chronic PD were collected from seven centers affiliated with the Socioeconomic Status on the Outcome of Peritoneal Dialysis (SSOP) Study. All demographic and laboratory data were recorded at baseline. Multivariate Cox regression was used to calculate the hazard ratio (HR) of CV and all-cause mortality with adjustments for recognized traditional and uremia-related CV factors.

**Results:**

There were no significant differences in baseline characteristics between patients with (n = 2193) and without (n = 71) UA measured. Each 1 mg/dL of increase in UA was associated with higher all-cause mortality with 1.05(1.00∼1.10) of HR and higher CV mortality with 1.12 (1.05∼1.20) of HR after adjusting for age, gender and center size. The highest gender-specific tertile of UA predicted higher all-cause mortality with 1.23(1.00∼1.52) of HR and higher CV mortality with 1.69 (1.21∼2.38) of HR after adjusting for age, gender and center size. The predictive value of UA was stronger in patients younger than 65 years without CV disease or diabetes at baseline. The prognostic value of UA as both continuous and categorical variable weakened or disappeared after further adjusted for uremia-related and traditional CV risk factors.

**Conclusions:**

The prognostic value of UA in CV and all-cause mortality was weak in PD patients generally, which was confounded by uremia-related and traditional CV risk factors.

## Introduction

Increased cardiovascular (CV) events have been extensively documented in patients with end-stage renal disease (ESRD) including peritoneal dialysis (PD) and hemodialysis(HD) population [1], [2]. CV events still accounts for approximately 40% of the annual mortality in dialysis patients [3]. Although numerous risk factors, categorized into traditional, uremic-related and non-traditional factors, have been recognized in recent years [4], [5], series of meta-analysis have not been able to demonstrate significant effect of targeting some of factors such as hyperlipidemia [6], hyperhomocystinemia [7], oxidative stress [8] and hyperphosphatemia [9] on outcomes in this high-risk patient group. Exploring novel and potentially modifiable risk factors for CV and all-cause mortality is therefore urgent.

Uric acid (UA), as one of novel risk factors, has been paid more attention in recent years. In general population, previous studies have shown that UA is closely associated with hypertension, coronary heart disease and chronic kidney disease (CKD) [10]–[12]. High UA also could independently predict CV events and mortality for ones with chronic diseases including CKD [13]–[15]. For dialysis population, a few studies from HD population indicated inconsistent relationship between UA and outcomes, that is, UA is negatively or ‘J-shaped’ related to all-cause or CV mortality [16]–[19]. There is no specific data on UA and outcomes for PD population yet.

In the present study, we aimed to explore associations of UA, all-cause and CV mortality in a large-scale multi-center PD cohort. The prognostic value of UA would be compared between patients≥65 years and <65years, with or without CV disease (CVD), diabetes and non-diabetes at baseline respectively. In addition, we determined whether associations of UA and outcomes would be changed after controlling for uremic-related factors(albumin, hemoglobin, residual renal function, phosphate etc) and traditional CV risk factors (hypertension, hyperlipidemia, obesity, diabetes, etc).

## Methods

This is an affiliated study with the Socioeconomic Status on the Outcome of Peritoneal Dialysis (SSOP) Study, which is a retrospective multi-center cohort study as described in detail in our previous paper [20]. The ethics committee of Peking University First Hospital approved this study.

### Centers enrollment

Centers which have professional PD doctors and PD nurses, and have well-developed databases of at least 3-years duration, recording baseline characteristics and follow-up data every 1∼3 months for each patient in our country participated this study voluntarily. Totally 9 centers were qualified, and 7 of them agreed to participate providing about 70 percent of all incident patients from 9 centers. Enrolled centers were from 5 provinces and located at 4 geographical regions (north, northeast, northwest, or east) in China. Data from each center have been collected within the strict quality control framework and further inspected and optimized to keep integrity and accuracy of the database. All study investigators and staff members completed a training program that taught them the methods and processes of the study. A manual of detailed instructions for data collection was distributed.

### Subjects selection

All the incident patients on chronic PD between the date of intact database creation and August 2011 were enrolled into this study. Each patient signed informed consent to agree their demographic and lab data to be used in future studies since they started PD therapy. All subjects began the PD program within one month after catheter implantation and were given lactate-buffered glucose dialysate with a twin-bag connection system (Baxter Healthcare, Guangzhou, China).

### Data collection

Demographic and clinical data including age, gender, body mass index (BMI), socioeconomic status (income and education level, living condition, etc), primary renal disease, the presence of cardiovascular disease (CVD) and diabetes mellitus (DM) were collected at baseline. Center size was also recorded according to number of enrolled patients of each center. CVD was recorded if one of the following conditions was present: angina, class III–IV congestive heart failure (NYHA), trandient ischemic attack, history of myocardial infarction or cerebrovascular accident and peripheral arterial disease [21].

Blood pressures were measured according to the guidelines presented in the Seventh Report of the Joint National Committee on Prevention, Detection, Evaluation and Treatment of High Blood pressure [22]. Patients took antihypertensive medications and performed the bag exchange as usual at their home on the morning of each clinic visit. A skilled nurse using a mercury sphygmomanometer measured brachial blood pressure in sitting position after they had rested for at least 10 minutes in a quiet and peaceful room. Systolic and diastolic blood pressure, and calculated mean arterial pressure during the first 3 months were averaged for at least three times of readings.

Biochemistry data including hemoglobin, serum albumin, UA, lipids spectrum, glucose, calcium, phosphate and intact parathyroid hormone (iPTH) were examined using an automatic Hitachi chemistry analyzer. The first testing was completed within one month of PD at the first visit, and then repeatedly once a month. The mean values in the first 3 months were calculated. The coefficient of variation of UA from multiple measurements was 5.3% for subjects from Peking University First Hospital but not recorded for those from other hospitals. Serum UA was measured by the uricase method using the same autoanalyzer. Serum high sensitive C-reactive protein (CRP) was measured by immune rate nephelometric analysis. Dialysis adequacy and residual renal function (RRF) were measured after one month of dialysis therapy. RRF was defined as the mean of residual creatinine clearance and residual urea clearance. Dialysis adequacy was defined as total Kt/V and total creatinine clearance. Corrected calcium was calculated by standard equation: Corrected calcium = serum total calcium+0.02*(40-serum albumin in g/L).

### Definition of outcome event

The outcomes were defined as cardiovascular and all-cause death. The cardiovascular death was defined as death due to myocardial infarction, congestive heart failure, cerebral bleeding, cerebral infarction, arrhythmia, peripheral arterial disease, and sudden death. In all analysis, we censored follow-up at trandferring to HD, loss to follow-up, renal trandplantation, or the end of the study (November 1, 2011).

### Statistical analysis

Continuous data were presented as mean with SDs except for CRP and RRF, which were presented as median (interquartile range) because of a high skew. Categorical variables were presented as proportions. Patients' data were compared by using the t-test or ANOVA F-test for normally distributed continuous variables, chi-square test for categorical variables, and Mann-Whitney U test for skewed continuous variables. UA was trandformed into categorical variable by gender-specific tertiles or quartiles.

For determining associations of UA, CV and all-cause mortality, UA as continuous variable was first examined in Cox regression models after adjusting for age, gender and center size (model 1) for all participants, and then in subgroups such as patients≥65 years or <65 years, with or without CVD, with or without DM respectively. Next, we explored whether associations of UA and CV/all-cause mortality in all participants were confounded by traditional and uremia-related CVD factors. Uremia-related factors such as serum albumin, hemoglobin, phosphate, RRF and CRP (model 2), and additional traditional CV factors such as BMI, the history of diabetes or CVD, mean arterial pressure and LDL cholesterol(model 3) were constructed respectively. For these examinations, UA was also considered as categorical variable by gender-specific tertiles or quartiles respectively but only gender-specific tertiles of UA was shown in the context since similar linear trends were indicated. Gender was not included as the adjusted variable if UA is examined as the gender-specific variable.

We reported the multivariable adjusted hazards ratios (HRs) with 95% CIs. All probabilities were two-tailed, and the level of significance was set at 0.05. Statistical analysis was performed by SPSS for Windows software version 13.0 (SPSS Inc., Chicago, IL).

## Results

### Baseline characteristics and follow-up

A total of 2264 PD patients were collected, with a mean age of 58.1±15.5 years, BMI of 22.9±3.6 kg/m^2^; 37.7% were diabetic and CVD was present in 41.5% of subjects at baseline. Chronic glomerulonephritis was the most common cause of ESRD (34.4%), followed by diabetic nephropathy (29.3%) and hypertensive nephropathy (15.5%). There were 71 of 2264 patients without UA values at baseline. The mean age, BMI, MBP, serum lipids, distribution of primary renal disease, CVD history, prevalence of DM were not significantly different between those who had measured UA and those who did not (2193 patients) (*P*>0.05). A total of 80 patients with inactive solid organ tumors at baseline were not excluded since their UA values were comparable to the remainders, and linear trends of UA and outcomes were not changed when they were excluded. Thereafter, 2193 patients were included in the final analysis.

The median follow-up time was 26.5(13.6∼43.6) months. At the end of study, of 586 (26.7% of 2193) patients who died, 231 cases (39.4%) were due to CVD, 140 cases (23.8%) were due to infection, and other causes of death included malignancy (11.9%), gastrointestinal bleeding (4.3%), severe malnutrition (4.8%), miscellaneous (5.1%), and undefined (10.6%). Of 231 patients who died from CVD, the leading cause was myocardial infarction (52 cases, 22.5%), followed by congestive heart failure (19.0%), cerebral bleeding (14.3%), cerebral infarction (12.9%), sudden death (9.5%), arrhythmia (4.8%), peripheral arterial disease (1.3%), and undefined causes (15.1%).

### UA and clinic characteristics

**Figure 1:**
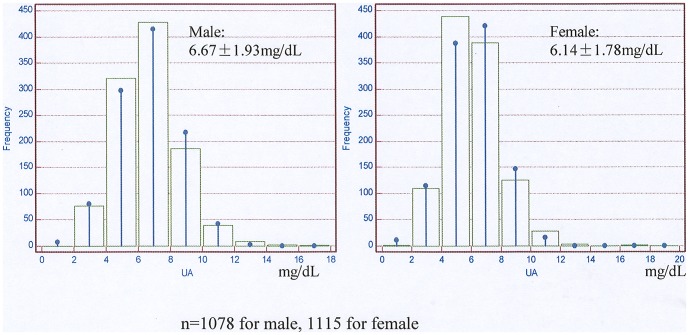
The distribution chart of serum uric acid.

The mean values of UA were 6.41±1.87 mg/dL for the whole cohort. The normal distribution of UA in male and female was shown respectively in **Fig. 1**. The clinical characteristics and biochemistry data of patients by gender-specific tertiles of UA were represented in **Table 1**. Patients with higher UA were more likely to be younger and obese. The prevalence of CVD was highest but diabetes was lowest in high tertile group. Systolic and diastolic blood pressure, serum albumin, urea nitrogen, creatinine, phosphate, and parathyroid hormone levels increased, but corrected calcium, hemoglobin and total Kt/V decreased in the middle/high tertiles. Serum CRP, triglycerides, total cholesterol, HDL and LDL cholesterol, total Ccr and RRF levels were not significantly different between groups (*P*>0.05).

**Table 1:** Clinical characteristics and biochemistry data of patients by gender-specific tertiles of UA.

Variables	Tertile 1M: 2.09∼5.79 mg/dL FM: 1.74∼5.37 mg/dL	Tertile 2M: 5.80∼7.38 mg/dL FM: 5.38∼6.65 mg/dL	Tertile 3M: 7.39∼16.7 mg/dL FM: 6.66∼8.08 mg/dL	*P* values&
N	731	731	731	*—*
Age, yrs	61.9±15.1***	57.7±15.4ΔΔΔ	54.7±15.3###	<0.001
Male (%)	49.2	49	49.2	0.99
BMI, Kg/m^2^	22.4±3.5***	23.3±3.6	23.1±3.7###	<0.001
Diabetes (%)	40.2	40.6ΔΔ	32.8##	0.003
CVD history (%)	34.8**	42.7	46.6###	<0.001
Systolic blood pressure, mmHg	133.5±22.5**	137.8±18.9	138.7±16.5###	<0.001
Diastolic blood pressure, mmHg	78.4±12.9**	80.5±12.9	82.1±11.2##	<0.001
Triglycerides, mmol/L	1.84±1.28	1.94±1.29	1.87±1.08	0.23
Total cholesterol, mmol/L	4.92±1.27	4.97±1.28	4.82±1.23	0.08
HDL cholesterol, mmol/L	1.16±0.38	1.13±0.35	1.14±0.37	0.2
LDL cholesterol, mmol/L	2.71±0.89	2.71±0.96	2.69±0.93	0.97
Albumin, g/L	33.9±5.4***	35.6±5.1ΔΔ	36.5±5.2###	<0.001
Hemoglogin, g/dL	103.6±19.5	104.2±18.6	101.7±19.8	0.04
UA, mg/dL	4.5±0.8***	6.3±0.5ΔΔΔ	8.4±1.4###	<0.001
Urea nitrogen, mmol/L	17.7±6.3***	21.0±6.3ΔΔΔ	23.1±7.1###	<0.001
Creatinine, umol/L	621.8±242.8***	695.0±246.9ΔΔΔ	728.9±274.5###	<0.001
Corrected calcium, mmol/L	2.29±0.25	2.29±0.25ΔΔΔ	2.25±0.24###	<0.001
Parathyroid hormone, pg/ml	157.8 (74.5, 318.9)	163.5(75.9, 314.8)Δ	196.8(91.8, 345.7)#	0.02
Phosphate, mmol/L	1.4±0.4***	1.6±0.4ΔΔΔ	1.7±0.5###	<0.001
CRP, mg/L	3.1(1.2, 6.9)	2.6(1.0, 7.8)	2.8(1.0, 7.5)	0.32
Total Kt/V	2.19±0.65***	2.01±0.60	1.95±0.63###	<0.001
Total Ccr, L/w/1.73m2	76.2±30.9	73.5±33.6	73.3±30.3	0.23
RRF, ml/min	3.1(1.4, 5.2)	3.2(1.6, 5.2)	3.7(1.8, 5.8)	0.16

Abbreviations: M, male; FM, female; UA, uric acid; BMI, body mass index; CVD, cardiovascular disease; CRP, C-reactive protein; Ccr, creatinie clearance; RRF, residual renal function.

^&^ P for comparisons among tertiles.

*P<0.05,

P<0.01,

P<0.001 for Tertile 1 vs Tertile 2.

P<0.05,

P<0.01,

P<0.001 for Tertile 2 vs Tertile 3.

^#^ P<0.05,

^##^ P<0.01,

^###^ P<0.01 for Tertile 1 vs Tertile 3.

### The association between UA and outcome

**Figure 2:**
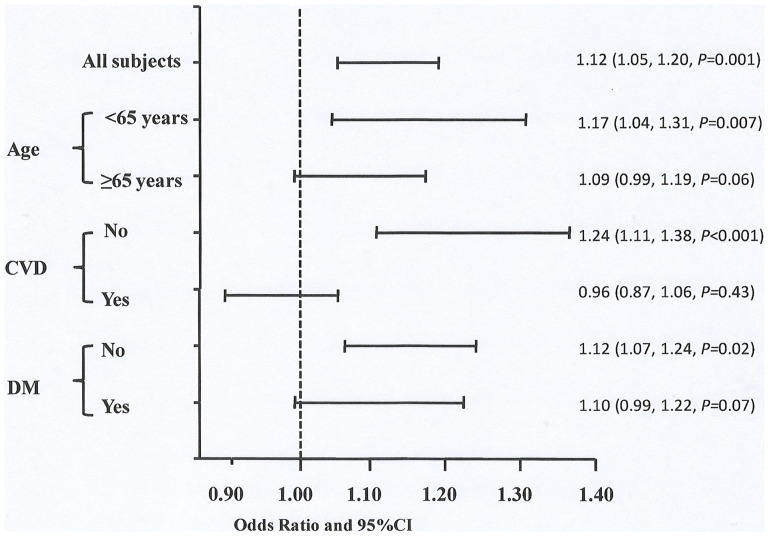
Risk of CVD mortality in all subjects and subgroups. Subgroups were divided by age ≥65 years or <65 years, with or without CVD or DM at baseline. All models are adjusted for age, gender and center size. Abbreviations: UA, uric acid; CDV, cardiovascular disease; DM, diabetes.

The associations between UA and outcomes were analyzed. First, UA was examined as a continuous variable. Each 1 mg/dL of increase in UA was associated with higher all-cause mortality with 1.05(1.00∼1.10) of HR(*P* = 0.05) and higher CV mortality with 1.12 (1.05∼1.20) of HR(*P* = 0.001) after adjusting for age, gender and center size **(** **Tables 2** **and** **3** **)**. We further divided patients into subgroups, i.e.age≥65 years and <65years, with and without CVD, DM and non-DM respectively. For CV mortality rather than all-cause mortality, the prognostic value of UA was significant in low-risk groups such as patients with age<65 years, without CVD or DM at baseline rather than in their counterpars respectively (**Figs. 2** **and** **3**). Next, UA was examined as categorical variable by gender-specific tertiles and quartiles (data not shown for the latter). The highest gender-specific tertile of UA predicted higher all-cause mortality with 1.23(1.00∼1.52) of HR(*P* = 0.04) and higher CV mortality with 1.69 (1.21∼2.38) of HR(*P* = 0.002) after adjusting for age, gender and center size compared to low tertile of UA. However, the associations of UA and CVD/all-cause mortality weakened after further adjusted for uremia-related factors including serum albumin, hemoglobin, phosphate, and CRP, and disappeared with additional adjustement for traditional CV factors such as CVD history, DM, body mass index, and LDL cholestrol **(** **Table 2** **and** **3** **)**.

**Figure 3:**
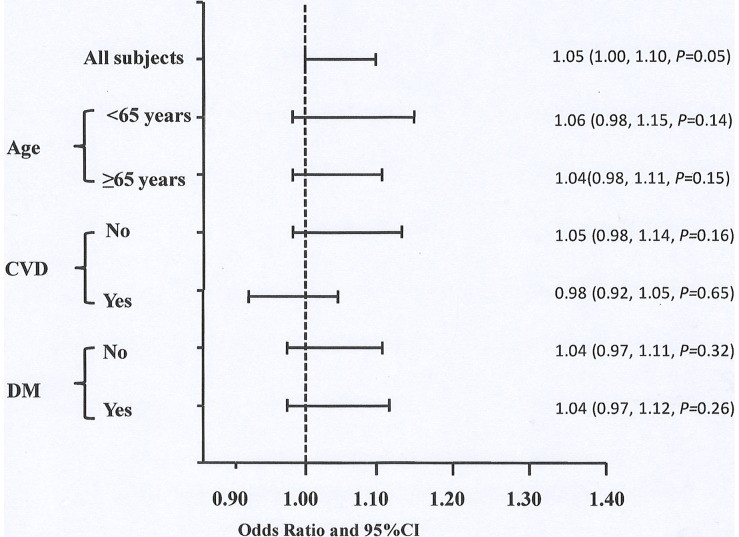
Risk of all-cause mortality in all subjects and subgroups. Subgroups were divided by age ≥65 years or <65 years, with or without CVD or DM at baseline. All models are adjusted for age, gender and center size. Abbreviations: UA, uric acid; CDV, cardiovascular disease; DM, diabetes.

**Table 2:** The prognostic values of UA as continuous or categorical variable for all-cause mortality.

Subjects	Age, gender-adjusted in Model 1		Multivariate-adjusted in Model 2		Multivariate-adjusted in Model 3	
	Hazard ratio (95% CI)	*P*	Hazard ratio (95% CI)	*P*	Hazard ratio (95% CI)	*P*
UA (per 1 mg/dL increase)	1.05(1.00∼1.10)	0.05	1.05(0.98∼1.12)	0.15	1.05(0.96∼1.14)	0.34
Gender-specific tertiles of UA						
Tertile 1	Reference		Reference		Reference	
Tertile 2	1.09(0.89∼1.33)	0.41	1.26 (0.97∼1.65)	0.09	1.23 (0.90∼1.70)	0.19
Tertile 3	1.23 (1.00∼1.52)	0.04	1.30 (0.98∼1.75)	0.07	1.21 (0.85∼1.73)	0.3

Abbreviation: UA, uric acid; CI, confidence interval.

Model 1: age,center size-adjusted, gender-adjusted only UA as continuous variable.

Model 2: age,residual renal function, serum albumin, hemoglobin,phosphate, C-reactive protein and center size-adjusted, gender-adjusted only UA as continuous variable.

Model 3: age, residual renal function, serum albumin, hemoglobin,phosphate, C-reactive protein, the histroy of cardiovascular disease,diabetes, body mass index, mean arterial pressure, LDL cholestrol and center size-adjusted, gender-adjusted only UA as continuous variable.

**Table 3:** The prognostic values of UA as continuous or categorical variable for cardiovascular mortality.

Subjects	Age, gender-adjusted in Model 1		Multivariate-adjusted in Model 2		Multivariate-adjusted in Model 3	
	Hazard ratio (95% CI)	*P*	Hazard ratio (95% CI)	*P*	Hazard ratio (95% CI)	*P*
UA (per 1 mg/dL increase)	1.12(1.05, 1.20)	0.001	1.05(0.95, 1.17)	0.35	1.04(0.89, 1.20)	0.65
Gender-specific tertiles of UA						
Tertile 1	Reference		Reference		Reference	
Tertile 2	1.56(1.13, 2.15)	0.007	1.48(0.96, 2.29)	0.08	1.29(0.75, 2.23)	0.35
Tertile 3	1.69(1.21, 2.38)	0.002	1.50(0.93, 2.41)	0.09	1.35(0.74, 2.46)	0.33

Abbreviation: UA, uric acid; CI, confidence interval.

Model 1: Age, center size-adjusted, gender-adjusted only UA as continuous variable.

Model 2: Age, residual renal function, serum albumin, hemoglobin,phosphate, C-reactive protein and center size-adjusted, gender-adjusted only UA as continuous variable.

Model 3: Age, residual renal function, serum albumin, hemoglobin,phosphate, C-reactive protein, the history of cardiovascular disease, diabetes, body mass index, mean arterial pressure, LDL cholestrol and center size-adjusted, gender-adjusted only UA as continuous variable.

## Discussion

In contrast to previous studies on HD patients showing a negative or ‘J-shaped’ relationship between UA and mortality [16]–[19], the present PD study did ont indicate similar trends between UA and CV or all-cause mortality. One may suspect that it is due to that the mean UA values in this cohort is quite different. This hypothesis is easily denied since the mean UA values(6.4 mg/dL) for our participants were very close to those reported in HD patients [16]–[19], [23]. It was also hypothesized that the inverse association of UA and outcome previously reported is confounded by nutrition status as indicated from DOPPS data [19]. From our data, although serum UA was also closely associated with higher body mass index, serum albumin, creatinine, phosphorous and better residual renal function, we could not observe a similar trend to HD patients.

The inconsistent trend of UA and outcomes was more likely to be explained by its dual effects on CV outcomes. Excess UA is closely related to components of metabolic syndrome, endothelial dysfunction, inflammation, oxidative stress and activated renin-angiotensin-aldosterone system in general population and patients with CKD [16], [24]–[29]. On the other hand, both in vitro and in vivo studies have shown UA to be a powerful free radical scavenger in humand and could be expected to offer a number of benefits within the cardiovascular system [30], [31].Therefore, the final trend for the association of UA and outcomes for a specific population might depend on the balance between the protective and toxic effects of UA. In addition, hyperuricaemia is significantly associated with the rate of decline of RRF [32], and RRF play a critical role in predicting CV events and all-cause death in PD population [33], which might partly contribute to the weakly negative associations of hyperuricaemia and outcomes for our PD patients.

Another interesting finding from our data is that the prognostic value of UA in CV mortality only existed in relatively low-risk patients including ones younger than 65 years, without CVD or DM at the start of PD therapy. This phenomenon has been indicated in previous data. For example, UA levels at either extremes predicted higher risk for cardiovascular mortality in general population, which was stronger in subgroups without DM, hypertension, coronary heart disease, stroke, heart failure and CKD [34]. The association between UA and renal function decline was more obvious in subgroups without hypertension and DM from a Chinese population [35]. Inverse associations of serum UA and morbidity of acute ischemic stroke were observed only in non-DM hemodialysis patients [36]. The potential cause for this phenomena is not clear but it might be relevant to concomitant confounders for CV mortality muting the association of UA and outcome in high-risk subjects. This finding also call us to pay more attention to low-risk subjects with elevated UA value.

Our data further indicated that the association of UA and CV/all-cause mortality was not independent, but rather related to concomitant uremia-related and traditional CV risk factors for PD population. This finding is in accordance with previous data from general population [37]–[40] and patients with CKD [41], [42], showing that independent role of UA weakened or abolished after controlling for some traditional or non-traditional factors. Recently, Chen et al also showed that the association between UA and acute ischemic stroke was confounded by demographic characteristics and malnutrition-microinflammation syndrome in Chinese HD patients [36]. Therefore, whether hyperuricemia represents a marker or a cause for CV events and mortality is still not clear for CKD population [43], [44]. Interventional studies should focus on this area to demonstrate if CKD patients would benefit from interventions lowering the elevated UA value.

Our large-scale multi-center cohort study gave us a valuable chance to observe the weak association of UA and CV/all-cause mortality in PD population for the first time. All participants were enrolled from ‘core’ PD centers of medical school affiliated hospital, which ensure the integrity and accuracy of clinical data for statistical adjustments. Second, multiple measurements of baseline laboratory were averaged, leading to more reliable data. Only 3.1% of participants missing UA values is also a merit. By contrast, most studies only have one single-point measurement [13], [15]–[17], [19], [36] or relatively higher percentage of missing values [19].

This study also has several of limitations. First of all, for observational studies where associations do not prove causality, residual confounding cannot entirely be excluded. As a retrospective study, the quality of data collection must have been affected by many uncontrolled factors. There was even no information about the diuretics and/or allopurinol treatment. Smoking habits, alcohol consumption and other non-traditional CV factors were not entirely examined. Howerver, if confounding occurred, it would result in underestimation of the association but not change our main findings at all. In addition, we should be aware of the possibilities of ascertainment bias (totally 3.1% eligible patients were not included).

In conclusion, we suggested that high UA is weakly associated with CV and all-cause mortality for PD population. More large-scale PD cohort studies are needed to verify our findings. Whether high UA levels should be modified for PD patients as done for CKD patients is to be determined.
